# Ethnic and racial inequity and inequality in health and science: a call for action

**DOI:** 10.1016/j.eclinm.2021.100782

**Published:** 2021-02-26

The COVID-19 pandemic has highlighted how different communities are affected by disease. More than at any time in the past these disparities are being exposed. During the first wave of COVID-19 in the UK (January to August, 2020), people of Black African background had the highest age-standardised COVID-19 mortality rates, which was five times higher than White British men; and during the second wave (September to December, 2020) people of south Asian ethnicity had a five times greater risk of mortality than White British people. The trend is similar in the USA. In proportion to the percentage of the population, African Americans are overrepresented in COVID-19 confirmed deaths. Against the backdrop of an infectious disease responsible for disproportionate mortality, the news of several effective COVID-19 vaccines should be welcome news also for members of the most afflicted communities. A survey published in February 2021 by the US National Foundation for Infectious Diseases identified that only 49% of Black adults planned to be vaccinated, with many having varying levels of trepidation about ever receiving the vaccine. These findings are similar to the Kaiser Family Foundation analysis and polling from January 2021, which finds that general vaccine uptake rates for Black people across many states are substantially lower than those for White people. The UK Household Longitudinal Study based at the University of Essex in November 2020, showed a similar trend of vaccine hesitancy among ethnic minorities. Vaccine hesitancy was found to be at 72% for Black people and 42% for people from Pakistani and Bangladeshi backgrounds.

In recognition of this gap between interventional need and community acceptance, the UK Scientific Advisory Group for Emergencies SAGE, which advises the UK government on scientific and healthcare matters, stated in their executive summary in December 2020 on factors affecting minority vaccine uptake: “Trust is particularly important for Black communities that have low trust in healthcare organisations and research findings due to historical issues of unethical healthcare research”. Such issues of unethical healthcare refer to a number of historical offensive incidences and acts in modern healthcare where those trusted with the responsibility to provide for those were often heinously misused. These concerns persist among minority groups globally. Conducted in the USA from the 1930s to 1970s the Tuskegee syphilis study, frequently referenced by ethnic minorities in various countries, involved acts of gross ethical misconduct and the withholding of appropriate treatment. From at least as far back as the 1700s, incidents of experimentation on enslaved for medical gain such as the work conducted by physicians such as J. Marion Sims remain present in the collective conscience.

While these crimes remain in the forefront of the minds of many people of colour, the substantial history of contributions by people of colour to medicine are not popularised. Individuals such as Jane Cooke Wright and Ernest Just's pioneering work in sickle cell anaemia and cancer treatments, respectively are examples of how minorities influenced our understanding of medicine and treatment. The history of deadly infectious diseases in the USA can be bookended by the contributions of an enslaved African man named Onesimus who instructed Bostonian doctors in variolation in the 1700s and Dr Kizzmekia Corbett in 2020 to the present who is leading the development of the NIH-Moderna COVID-19 vaccine. These and numerous contributions by ethnic minorities in the advancement of medical science should be popularised and discussed as part of the foundation of establishing minority relationship and trust with medicine. In order to make this a reality, medical authorities must make efforts to speak to minority communities on their terms and on a more consistent basis. Medical research must also seek to better represent minority individuals via clinical trials and study participation.

As part of *The Lancet's* Racial and ethnic equality—time for concrete action, *EClinicalMedicine* is curating a virtual collection on the topic of “Ethnic and racial inequity and inequality in health”. Our aim for this collection is to feature content that highlights racial disparities in communities, healthcare systems, and science globally. By highlighting gaps in healthcare and taking a critical look at our society, we aim to illuminate and demonstrate that change is needed to build fairer societies for all.

This collection focuses on evidence-based work which addresses and reveals disparities in disease progression and mortality between people of different ethnicities and races in the same communities. Similarly, we recognise the need to highlight work which assesses racial differences and discrimination in access and adherence to treatment alongside the systemic and organisational nature of healthcare systems.

Contributions are invited to address racial inequities across all speciality areas of medicine, interventions, healthcare decolonisation, intersections of ethnicity and gender, systemic racism in healthcare, ethical use of digital health data, diversity in healthcare staffing and decision making, mental health impact of inequality, interactions of genetics and socioeconomics in disparities, and race, ethnicity, and interactions with frontline healthcare workers. In order to affect change and assure the best quality of life for all we must be confident in placing a spotlight on the imbalances, injustice, and discrimination in our communities.*EClinicalMedicine*

